# Knowledge, attitudes, and practices of human papillomavirus and self-sampling among adult women: a cross-sectional study

**DOI:** 10.3389/fpubh.2024.1377343

**Published:** 2024-06-04

**Authors:** Juan Shao, Huihui Ke, Cui Jiang, Huanmei Sun, Hongyu Han, Jianlong Zhu, Li Chen, Yingchen Wang, Jiashi Gu, Yingchun Duan

**Affiliations:** Department of Obstetrics and Gynecology, Shanghai Pudong Hospital, Fudan University Pudong Medical Center, Shanghai, China

**Keywords:** human papillomavirus, knowledge, attitude, practice, cross-sectional survey, self-sampling

## Abstract

**Background:**

This study aimed to investigate the knowledge, attitude, and practice (KAP) of human papillomavirus (HPV) and self-sampling among adult women.

**Methods:**

The cross-sectional, questionnaire-based study included adult women at Shanghai Pudong Hospital from October 14, 2022, to March 31, 2023. The questionnaire contained demographic information, knowledge, attitude and practice dimensions. Factors associated with KAP and self-sampling were identified by multivariate logistic regression.

**Results:**

A total of 1843 valid questionnaires were collected. The average knowledge, attitude, and practice score was 10.09 ± 5.60, 26.76 ± 3.80, and 6.24 ± 2.20, respectively. Urban residents (estimate = 0.705, *p* < 0.001), suburban residents (estimate = 0.512, *p* < 0.001), as well as individuals with undergraduate degrees and higher (estimate = 0.535, *p* < 0.001), were associated with good knowledge, while individuals lacking a history of HPV infection (estimate = −0.461, *p <* 0.001) and married individuals (estimate = −0.185, *p* < 0.001) were less likely to have good knowledge. Higher knowledge scores (estimate = 0.087, *p <* 0.001) and individuals with undergraduate education and above (estimate = 1.570, *p <* 0.001) were associated with a positive attitude. Being married (estimate = 0.291, *p =* 0.049) was associated with good practice, whereas not engaging in sexual activity (estimate = −0.959, *p <* 0.001) or lacking a history of HPV infection (estimate = −0.499, *p =* 0.011) were associated with unfavorable practices. Minorities (OR = 2.787, *p* = 0.038) and individuals with multiple sexual partners (OR = 2.297 for two partners, OR = 2.767 for three or more partners, *p* = 0.020 and *p* = 0.022) were positively associated with self-sampling. However, higher knowledge (OR *=* 0.952, *p =* 0.026) and attitude scores (OR *=* 0.929, *p =* 0.015) were negatively associated with self-sampling.

**Conclusion:**

Demographic and behavioral factors significantly influenced KAP scores and self-sampling behaviors regarding HPV. Urban residency, higher education levels, positive attitudes, and minority status correlated with favorable outcomes, while factors like marriage and lack of sexual activity were associated with less favorable practices.

## Introduction

Human papillomavirus (HPV) is a prevalent cause of vulvar., vaginal, and cervical cancers, with approximately 50–80% of sexually active women acquiring an HPV infection at some stage of their lives (1, 2). Although some women may develop antibodies against HPV, these are detected in only a minority of infected individuals and offer modest protection against reinfection, which is unlikely to significantly contribute to viral clearance (3). Persistent infection with high-risk HPV strains, such as 16 and 18, poses a substantial risk of progressing to precancerous lesions, eventually evolving into invasive cancers (4). Primary prevention of HPV through prophylactic vaccination is crucial as it aims to prevent the initial infection with the virus, thereby reducing the risk of advancing to cancerous stages (5). This preventive strategy complements efforts focused on the early detection and treatment of HPV infections, which are vital in preventing their progression to advanced stages (6).

HPV testing is a valuable method for preventing cervical cancer due to its superior negative predictive value and higher sensitivity compared to cytology-based screening (7). It is particularly beneficial in identifying precancerous lesions and early-stage cancers (8). This enables longer screening intervals, which is especially advantageous in resource-limited settings (9). Recent meta-analyses showed that self-sampling significantly boosts cervical cancer screening participation, especially in low-income settings, with mail-to-all strategies being the most effective. However, further studies are needed to assess its economic and full-scale implementation viability (10–12). Given that many women are either underscreened or undergo screening too frequently (13, 14), the significance of self-sampling for HPV testing is underscored. Self-sampling, by improving screening rates and enabling timely detection of cervical abnormalities, empowers women to take control of their cervical health, overcoming barriers such as embarrassment, inconvenience, and limited healthcare access (11, 15).

HPV self-sampling testing in China has yielded mixed results in terms of acceptability. Studies have reported that 83.27% of participants were willing to choose self-sampling for cervical cancer screening (16), while another study found that only 12.5% of women had heard of and undergone self-sampling (17). These findings highlight regional and population-specific variations in the acceptability of HPV self-sampling in China. Thus, it is important to further investigate and understand the factors that influence the acceptability of HPV self-sampling in China to develop effective strategies for increasing screening participation rates.

This study aimed to assess the knowledge, attitude, and practice (KAP) of HPV and self-sampling among adult women in Shanghai, China, as KAP studies are crucial for understanding the current landscape and identifying areas for improvement, ensuring informed decision-making and promoting better healthcare outcomes (18). The results can potentially guide the development of policies and interventions aimed at promoting HPV prevention and early detection strategies.

## Methods

### Study design and participants

The cross-sectional, questionnaire-based study included adult women at Shanghai Pudong Hospital from October 14, 2022, to March 31, 2023. The study was approved by the Shanghai Pudong Hospital Medical Ethics Committee, and informed consent was obtained from all participants. Inclusion criteria were as follows: (1) Age ≧18 years old; (2) Voluntary participation, a full understanding of the research project, and the ability to sign informed consent. Exclusion criteria included: (1) Response time of ≤90 s; (2) Questionnaires with completely repeated answers for each item.

### Questionnaire and quality control

The questionnaire was developed by referring to relevant references (19, 20) and was administered on a small scale, with 30 copies distributed. The questionnaire exhibited acceptable internal consistency, as indicated by a Cronbach’s α coefficient of 0.707. The final questionnaire contained four dimensions: demographic information (including age, education, and other components), knowledge, attitude, and practice. The knowledge dimension comprised 10 questions. Questions 1 to 7 were presented with three response options: “correct,” “incorrect,” and “unclear.” These responses were scored as follows: 2 points were awarded for “correct” answers, 1 point for “unclear” responses, and 0 points for “incorrect” answers. Questions 8 to 10 were rated on a similar scale: 2 points for “understand,” 1 point for “partially understand,” and 0 points for “do not understand.” Participants determined responses based on personal assessments of understanding. The scores in this dimension ranged from 0 to 20. The attitude dimension consisted of 8 questions, which were scored using a five-point Likert scale (“strongly agree” = 5 points, “agree” = 4 points, “neutral” = 3 points, “disagree” = 2 points, and “strongly disagree” = 1 point). The scores for this dimension ranged from 8 to 40. The practice dimension included 8 questions, with a score of 2 assigned for “yes” responses and 0 for “no.” Two questions were not scored. The scores in this dimension ranged from 0 to 12.

Before the commencement of the research project, the department director coordinated the training of the questionnaire survey staff to ensure their readiness and qualification for the task. The questionnaire was designed on Wenjuanxing, a professional online questionnaire software platform provided by Changsha Ranxing Information Technology (China) (21). A link to the questionnaire was generated and distributed to potential participants through WeChat groups. Additionally, department heads coordinated with relevant personnel to organize training sessions. To facilitate the process, QR codes linked to the questionnaire were made available at various locations within the hospital, including clinics, wards, and physical examination stations. This method enabled patients to easily scan the codes and access the questionnaire online. Assistance was provided for patients who had queries regarding the questionnaire content, with on-site staff readily available to answer any questions. Staff meetings were scheduled every one to 2 weeks to monitor and ensure the quality of the questionnaire collection process. No incentive was offered for the completion of the questionnaire.

### Statistical analysis

All statistical analyses were performed using SPSS 26.0 (IBM, Armonk, NY, United States) and Stata 18.0. Continuous variables were evaluated based on their distributional shape. Symmetrical variables were expressed as the mean ± standard deviation. Skewed variables were summarized using the median and interquartile range. Group comparisons were conducted using the Kruskal-Wallis test. Categorical variables were presented as frequency (percentage). Multivariate logistic regression analyses were used to identify factors associated with good practice, positive attitude, and high knowledge levels.

Kernel regression was employed to model the relationship between the dependent variable and independent variables without assuming a predetermined form of the relationship. Specifically, we utilized the local constant estimator approach for point estimation. This nonparametric method does not impose a parametric structure on the data, allowing for more flexible modeling of complex relationships.

To assess the reliability of the estimated parameters and construct confidence intervals, a bootstrap method was implemented. We performed 100 bootstrap resamplings to estimate the distribution of the estimator. Each bootstrap sample was drawn with a replacement from the original dataset, and the local constant estimator was recalculated for each resampled dataset. A two-sided *p*-value less than 0.05 was considered statistically significant.

## Results

### Demographic characteristics and KAP scores among participants

In this study, a total of 1872 questionnaires were collected. After excluding 20 questionnaires with response times ≤90 s, eight questionnaires with all “A” choices in the KAP section, and one duplicate questionnaire, 1843 valid questionnaires remained for analysis. The demographic information and KAP scores are presented in Supplementary Table S1. The participants demonstrated an average knowledge score of 50.45% (10.09 ± 5.60 out of 20), an attitude score of 66.9% (26.76 ± 3.80 out of 40), and a practice score of 52% (6.24 ± 2.20 out of 12).

Significant variations were observed in knowledge, attitude, and practice scores according to place of residence (*p <* 0.001 for knowledge, *p* = 0.026 for attitude, *p* < 0.001 for practice), ethnicity (*p* = 0.021 for knowledge, *p* = 0.019 for practice), level of education (*p <* 0.001 across all), employment status (*p <* 0.001 across all), household income (*p <* 0.001 across all) and history of HPV infection (*p <* 0.001 for knowledge, *p* = 0.021 for attitude, *p* < 0.001 for practice). Marital status also significantly influenced the scores (*p <* 0.001 for knowledge and practice). Practice scores were notably affected by sexual life (*p <* 0.001). Family history of cervical cancer significantly impacted the scores (*p <* 0.001 for knowledge and *p* = 0.0008 for practice). Lastly, smoking history had a significant effect on knowledge scores (*p* = 0.013).

### Knowledge, attitudes, and practices towards HPV and self-sampling

The distribution of responses for each knowledge item showed varying understanding of HPV and self-sampling (Table 1). The highest accuracy rate was observed for a question related to the linkage between HPV infection and cervical cancer, with 71.35% of respondents answering correctly, reflecting substantial awareness. Conversely, only 4.83% of respondents correctly responded to a statement about the progression of persistent HPV infection, indicating a major gap in understanding the progression of the disease. Furthermore, comprehension of HPV self-sampling was notably low, with only 12.70% fully understanding the concept, suggesting a significant area for educational improvement. Responses also showed limited understanding of the categorization into high-risk and low-risk HPV types, with only 32.66% fully grasping this classification.

**Table 1:** Distribution of the responses for each knowledge item.

	Correct	Wrong	Unclear
Human papillomavirus (HPV) is the leading causes of vulvar., vaginal, and cervical cancer.	1,230 (66.74)	39 (2.12)	574 (31.14)
HPV infection can be transmitted through unprotected sexual activity.	1,289 (69.94)	80 (4.34)	474 (25.72)
HPV infection may remain asymptomatic even after several years of infection.	751 (40.75)	325 (17.63)	767 (41.62)
Prior to HPV examination, avoid vaginal douching, intravaginal medication for three days, sexual activity for 24 h, and menstrual periods.	1,247 (67.66)	25 (1.36)	571 (30.98)
HPV infection is associated with the development of cervical cancer.	1,315 (71.35)	32 (1.74)	496 (26.91)
HPV types 16 and 18 are the most common high-risk trains.	935 (50.73)	22 (1.19)	886 (48.07)
Persistent HPV infection will eventually develop into cervical cancer.	89 (4.83)	1,215 (65.93)	539 (29.25)

	Understand	Partially understand	Not understand
Do you understand that HPV types are classified into high-risk and low-risk categories?	602 (32.66)	743 (40.31)	498 (27.02)
Do you understand the risks associated with high-risk types of HPV?	688 (37.33)	678 (36.79)	477 (25.88)
Do you understand the concept of HPV self-sampling?	234 (12.70)	424 (23.01)	1,185 (64.30)

The survey results revealed notable attitudes towards HPV self-sampling and prevention methods (Table 2). A majority of respondents (56.43% strongly agreeing and 32.39% agreeing) expressed doubts about the ability of HPV self-sampling to ensure sample quality, and 16.23% (8.36% strongly agreeing and 7.87% agreeing) held reservations about the accuracy of results obtained through self-sampling. Concerns about potential sample contamination during the process of sending self-samples were also prevalent, with 16.22% strongly agreeing and 48.29% agreeing. However, there was a strong belief (55.24% strongly agreeing and 36.08% agreeing) that HPV testing can help prevent or decrease the chances of developing cervical cancer. Interestingly, only 44.16% of respondents (13.02% strongly agreeing and 31.14% agreeing) believed that HPV vaccination is crucial for preventing HPV infection. Regarding the convenience and comfort of self-sampling, only 24.31% of respondents considered it to be a convenient method, and 58.38% of them felt that self-sampling might be embarrassing. This indicates a significant barrier to self-sampling uptake due to comfort and privacy concerns.

**Table 2:** Distribution of the responses for each attitude item.

	Strongly agree	Agree	Neutral	Disagree	Strongly disagree
I have doubts about the ability of HPV self-sampling to ensure sample quality.	1,040 (56.43)	597 (32.39)	185 (10.04)	15 (0.81)	6 (0.33)
I believe that HPV testing can help prevent or decrease my chances of developing cervical cancer.	1,018 (55.24)	665 (36.08)	132 (7.16)	22 (1.19)	6 (0.33)
I have reservations about the accuracy of results obtained through self-sampling.	154 (8.36)	145 (7.87)	205 (11.12)	1,015 (55.07)	324 (17.58)
I believe that only individuals at high risk should undergo HPV testing.	271 (14.70)	701 (38.04)	557 (30.22)	280 (15.19)	34 (1.84)
I am concerned about potential sample contamination during the process of sending self-samples.	299 (16.22)	890 (48.29)	409 (22.19)	218 (11.83)	27 (1.47)
I believe that HPV vaccination is crucial for preventing HPV infection.	240 (13.02)	574 (31.14)	682 (37.00)	322 (17.47)	25 (1.36)
I consider self-sampling to be a convenient method for collecting samples.	134 (7.27)	314 (17.04)	643 (34.89)	647 (35.11)	105 (5.70)
I feel that self-sampling might be embarrassing for me.	261 (14.16)	815 (44.22)	534 (28.97)	204 (11.07)	29 (1.57)

The distribution of responses for each practice item is shown in Table 3. A majority of the participants (62.83%) reported having been tested for HPV. Among those tested, a very small fraction (5.97%) have used self-sampling, while a significant majority (88.50%) reported undergoing regular follow-up testing when tested. A high percentage of respondents (84.81%) expressed willingness to undergo regular HPV screening if they could collect their own samples, and 71.46% were interested in performing self-sampling themselves. Regarding recommendations, 70.05% would advise close female friends to consider self-sampling. For preferred locations for self-sampling, the majority chose hospitals (53.39%), followed by home (35.54%). Among the concerns regarding self-sampling, the main issues were the safety of sampling (18.39%), potential serum deterioration and contamination during mailing (18.34%), and reliability of results (16.93%). These findings suggest a general openness to self-sampling, tempered by significant concerns about the safety and efficacy of the process.

**Table 3:** Distribution of the responses for each practice item.

	Yes	No
Have you ever been tested for HPV?	1,158 (62.83)	685 (37.17)
If you have ever been tested for HPV, do you perform self-sampling?	110 (5.97)	1,048 (56.86)
If you were tested for HPV infection, were you able to undergo regular follow-up testing?	1,631 (88.50)	212 (11.50)
If you could collect your sample by yourself, would you like to undergo regular HPV screening?	1,563 (84.81)	280 (15.19)
Would you recommend self-sampling testing to your close female friends?	1,291 (70.05)	552 (29.95)
Would you like to perform self-sampling?	1,317 (71.46)	526 (28.54)

	Hospitals	Home	Clinics near your home	Other
The location of your desired to perform self-sampling.	984 (53.39)	655 (35.54)	148 (8.03)	26 (3.04)

	Safety of sampling	Difficulty of sampling operations	Possible serum deterioration and contamination during the mailing process	Reliability of the results	Difficulty understanding the meaning of the result	Others
If you are not willing to choose self-sampling, the reasons are (multiple choice)	339 (18.39)	297 (16.12)	338 (18.34)	312 (16.93)	153 (8.30)	80 (4.34)

### Factors influencing knowledge, attitudes, and practices towards self-sampling for HPV testing

The multivariate analysis showed that individuals living in urban areas had significantly higher odds of having good knowledge about HPV compared to those living in rural areas (estimate = 0.705, 95% CI: 0.599, 0.865, *p <* 0.001). Similarly, individuals living in suburban areas also had significantly higher odds (estimate = 0.512, 95% CI: 0.403, 0.649, *p <* 0.001). The level of education significantly affected the knowledge score, with individuals holding an undergraduate degree or above having a higher knowledge score (estimate = 0.535, 95% CI: 0.427, 0.667, *p <* 0.001) compared to those with primary education or below. Interestingly, marital status showed that married individuals were less likely to have good knowledge compared to unmarried individuals (estimate = −0.185, 95% CI: −0.249, −0.127, *p <* 0.001). Individuals without a history of HPV infection exhibited a significant decrease in the odds of possessing good knowledge (estimate = −0.461, 95% CI: −0.533, −0.368, *p <* 0.001) (Table 4).

**Table 4:** Multivariate logistic regression analysis of the factors associated with knowledge.

	Estimate	95% CI	*p*
Residence			
Rural	Ref.		
Urban	0.705	0.599, 0.865	<0.001
Suburban	0.512	0.403, 0.649	<0.001
Ethnicity			
Han	Ref.		
Minority	0.055	0.008, 0.092	0.009
Education			
Primary and below	Ref.		
Middle school	−0.556	−0.685, −0.420	<0.001
High school / technical secondary school	−0.226	−0.326, −0.113	<0.001
Junior college	0.579	0.468, 0.702	<0.001
Undergraduate and above	0.535	0.427, 0.667	<0.001
Employment status			
Employed	Ref.		
Unemployed	0.309	0.238, 0.404	<0.001
Retired	0.027	−0.019, 0.070	0.313
Freelance	0.056	−0.001, 0.114	0.048
Housewife	−0.062	−0.118, −0.000	0.070
Student	−0.130	−0.209, −0.077	0.001
Other	0.101	0.0573, 0.137	<0.001
Household income *per capita* (Yuan)			
<2000	Ref.		
2000–5,000	−0.066	−0.158,0.043	0.146
5,000–10,000	0.175	0.059, 0.265	0.005
10,000–20,000	0.390	0.313, 0.496	<0.001
>20,000	0.289	0.213, 0.378	<0.001
Marital status			
Unmarried	Ref.		
Married	−0.185	−0.249, −0.127	<0.001
Divorced / widowed	−0.111	−0.161, −0.061	<0.001
Sexual life			
Yes	Ref.		
No	−0.032	−0.107, 0.023	0.459
HPV family history			
Yes	Ref.		
No	−0.041	−0.080, 0.006	0.076
History of HPV infection			
Yes	Ref.		
No	−0.461	−0.533, −0.368	<0.001
Smoking status			
Never smoked	Ref.		
Former smoker	−0.075	−0.138, 0.002	0.034
Current smoker	−0.065	−0.118, −0.032	0.009

Additionally, an increase in knowledge score was significantly associated with a positive attitude (estimate = 0.087, 95% CI: 0.058, 0.121, *p <* 0.001). Individuals with undergraduate education and above demonstrated significantly higher odds of having a positive attitude compared to those with primary education and below (estimate = 1.570, 95% CI: 0.898, 2.411, *p <* 0.001) (Table 5).

**Table 5:** Multivariate logistic regression analysis of the factors associated with attitude.

	Estimate	95% CI	*p*
Knowledge score	0.087	0.058, 0.121	<0.001
Residence			
Rural	Ref.		
Urban	−0.073	−0.283, 0.212	0.582
Suburban	−0.156	−0.578, 0.432	0.561
Ethnicity			
Han	Ref.		
Minority	−0.119	−1.211, 0.968	0.829
Education			
Primary and below	Ref.		
Middle school	0.389	0.224, 0.599	<0.001
High school / technical secondary school	0.779	0.443, 1.192	<0.001
Junior college	1.169	0.665, 1.801	<0.001
Undergraduate and above	1.570	0.898, 2.411	<0.001
Employment status			
Employed	Ref.		
Unemployed	0.087	0.010, 0.226	0.149
Retired	0.175	0.020, 0.454	0.146
Freelance	0.263	0.029, 0.683	0.147
Housewife	0.358	0.045, 0.914	0.138
Student	0.434	0.041, 1.135	0.152
Other	0.535	0.070, 1.374	0.140
Household income *per capita* (Yuan)			
<2000	Ref.		
2000–5,000	0.159	0.017, 0.305	0.055
5,000–10,000	0.319	0.035, 0.608	0.054
10,000–20,000	0.478	0.052, 0.913	0.055
>20,000	0.637	0.069, 1.217	0.055
Marital status			
Unmarried	Ref.		
Married	−0.268	−0.786, 0.127	0.207
Divorced / widowed	−0.497	−1.554, 0.244	0.239
Sexual life			
Yes	Ref.		
No	0.037	−0.620, 0.633	0.922
HPV family history			
Yes	Ref.		
No	−0.671	−2.465, 0.148	0.343
History of HPV infection			
Yes	Ref.		
No	−0.348	−0.702, 0.066	0.127
Smoking status			
Never smoked	Ref.		
Former smoker	−0.348	−0.934, 0.047	0.183
Current smoker	−0.735	−1.836, 0.102	0.146

Furthermore, higher knowledge (estimate = 0.109, 95% CI: 0.094, 0.128, *p <* 0.001) and attitude scores (estimate = 0.085, 95% CI: 0.066, 0.114, *p <* 0.001) were significantly associated with good practice. Married individuals (estimate = 0.291, 95% CI: −0.034, 0.520, *p =* 0.049) had significantly higher odds of good practice compared to those who were unmarried. Not engaging in sexual activity (estimate = −0.959, 95% CI: −1.325, −0.514, *p <* 0.001) and lacking a history of HPV infection (estimate = −0.499, 95% CI: −0.879, −0.151, *p =* 0.014) were significantly associated with unfavorable practices (Table 6).

**Table 6:** Multivariate logistic regression analysis of the factors associated with practice.

	Estimate	95% CI	*p*
Knowledge score	0.109	0.094, 0.128	<0.001
Attitude score	0.085	0.066, 0.114	<0.001
Residence			
Rural	Ref.		
Urban	0.028	−0.089, 0.158	0.688
Suburban	−0.015	−0.232, 0.277	0.913
Ethnicity			
Han	Ref.		
Minority	0.529	0.075, 1.163	0.074
Education			
Primary and below	Ref.		
Middle school	−0.057	−0.203, 0.118	0.534
High school / technical secondary school	−0.119	−0.399, 0.193	0.475
Junior college	−0.143	−0.585, 0.275	0.549
Undergraduate and above	−0.205	−0.797, 0.385	0.517
Employment status			
Employed	Ref.		
Unemployed	−0.049	−0.127, 0.037	0.279
Retired	−0.127	−0.277, 0.006	0.101
Freelance	−0.224	−0.432, −0.003	0.057
Housewife	−0.262	−0.561, 0.004	0.100
Student	−0.284	−0.634, 0.070	0.150
Other	−0.434	−0.836, −0.019	0.054
Household income *per capita* (Yuan)			
<2000	Ref.		
2000–5,000	0.067	−0.073, 0.200	0.387
5,000–10,000	0.158	−0.092, 0.461	0.262
10,000–20,000	0.101	−0.178, 0.514	0.608
>20,000	0.181	−0.277, 0.727	0.480
Marital status			
Unmarried	Ref.		
Married	0.291	−0.034, 0.520	0.049
Divorced / widowed	0.216	−0.358, 0.651	0.444
Sexual life			
Yes	Ref.		
No	−0.959	−1.325, −0.514	<0.001
HPV family history			
Yes	Ref.		
No	0.087	−0.678, 0.811	0.815
History of HPV infection			
Yes	Ref.		
No	−0.499	−0.879, −0.151	0.014
Smoking status			
Never smoked	Ref.		
Former smoker	0.200	−0.156, 0.524	0.232
Current smoker	0.388	−0.233, 1.070	0.213

Moreover, a higher knowledge score (OR = 0.952, 95% CI: 0.911–0.994, *p =* 0.026) and a higher attitude score (OR = 0.929, 95% CI: 0.876–0.986, *p =* 0.015) were inversely associated with self-sampling for HPV testing. Belonging to a minority ethnic group was associated with a higher likelihood of self-sampling (OR = 2.787, 95% CI: 1.056–7.353, *p =* 0.038). Having more sexual partners was associated with a greater likelihood of self-sampling, with individuals who had two sexual partners (OR = 2.297, 95% CI: 1.137–4.639, *p =* 0.020) and those with three or more partners (OR = 2.767, 95% CI: 1.158–6.612, *p =* 0.022) showing higher odds than those with just one partner (Table 7).

**Table 7:** Multivariate logistic regression analysis of factors associated with self-sampling for HPV testing among the participants.

Variables	Multivariate analysis	
	OR (95%CI)	*p*
Knowledge score	0.952 (0.911 0.994)	0.026
Attitude score	0.929 (0.876 0.986)	0.015
Age (years old)		
18–30	Ref.	
30–35	0.978 (0.497 1.922)	0.948
35–40	1.838 (0.940 3.594)	0.075
40–45	0.611 (0.254 1.468)	0.270
45 and above	1.114 (0.522 2.376)	0.780
Residence		
Rural	Ref.	
Urban	0.990 (0.554 1.768)	0.972
Suburban	0.767 (0.420 1.402)	0.389
Ethnicity		
Han	Ref.	
Minority	2.787 (1.056 7.353)	0.038
Education		
Primary and below	Ref.	
Middle school	0.355 (0.082 1.533)	0.165
High school / technical secondary school	0.344 (0.076 1.566)	0.168
Junior college / undergraduate	0.248 (0.054 1.127)	0.071
Postgraduate and above	0.168 (0.026 1.086)	0.061
Household income *per capita* (Yuan)		
<2000	Ref.	
2000–5,000	0.745 (0.282 1.967)	0.553
5,000–10,000	0.513 (0.188 1.399)	0.192
10,000–20,000	0.436 (0.147 1.293)	0.134
>20,000	0.281 (0.078 1.019)	0.053
The number of sexual partners you have had		
1	Ref.	
2	2.297 (1.137 4.639)	0.020
≥3	2.767 (1.158 6.612)	0.022
Smoking status		
Never smoked	Ref.	
Former smoker	1.896 (0.788 4.566)	0.153
Current smoker	0.775 (0.165 3.632)	0.746

## Discussion

In the study, participants scored an average of 50.45% in knowledge, 66.9% in attitude, and 52% in practice regarding HPV and self-sampling. Significant variations in knowledge, attitudes, and practices were observed based on demographic factors such as age, education level, employment status, marital status, sexual activity, history of HPV infection, and ethnicity. This highlights the need for targeted educational interventions to improve knowledge and promote positive attitudes and practices related to HPV and self-sampling, particularly focusing on rural populations, individuals with lower education levels, and those with limited understanding of HPV concepts.

The participants in this study and a similar study in Brazil demonstrated a lack of knowledge regarding HPV and self-sampling, with average knowledge scores of 50.45 and 51.79%, respectively (22). This study highlighted the highest levels of knowledge among participants regarding the link between HPV and vulvar., vaginal, and cervical cancer (66.74%) and the transmission of HPV through unprotected sexual activity (69.94%). Significant knowledge gaps were observed in understanding the connection between persistent HPV infection and cervical cancer (4.83%) as well as HPV self-sampling (12.70%). Another study conducted in Dhaka district, Bangladesh, revealed that although a high percentage of women were aware of cervical cancer (87%), only a small proportion knew about HPV as a risk factor (13%) (23). Similarly, in a study conducted in Romania, 69.2% of women were aware of HPV, but their knowledge was limited (24). These findings highlight the need for comprehensive educational interventions to improve knowledge and awareness about HPV and its associated risks, particularly in relation to persistent HPV infection and self-sampling.

The study showed that a significant proportion of participants (56.43% strongly agreed and 32.39% agreed) had doubts about the ability of HPV self-sampling to ensure sample quality. Additionally, 16.23% expressed reservations about the accuracy of self-sampling results. Concerns about sample contamination during the process of sending self-samples were also prevalent, with 16.22% strongly agreeing and 48.29% agreeing. On the other hand, in studies conducted in South Africa and Chengdu, China, 57.7 and 42.32% of participants, respectively, expressed willingness to undergo future screening if allowed to self-sample (19, 20), indicating a more positive attitude towards HPV self-sampling. Further, in a study conducted in rural indigenous communities in Guatemala, there was significant acceptance and willingness to use self-collection for HPV testing. In one community, 94% of the age-eligible participants completed self-collection, and in another community, 53% of participants chose self-collection (25). The varying attitudes towards HPV self-sampling across regions may indicate differences in health literacy, cultural norms, trust in medical procedures, and access to healthcare. This suggests a need for comprehensive education and awareness efforts to address doubts and concerns, emphasizing the effectiveness and reliability of HPV self-sampling.

In this study, factors associated with good knowledge about HPV and self-sampling included living in urban or suburban areas, higher education levels, unmarried status, and a history of HPV infection. Consistently, higher education has been identified as a key factor linked to better knowledge about HPV in the general population (22, 24, 26). Interestingly, it seems that unique factors contribute to the understanding of HPV-related topics in specific demographic groups. A study conducted in Turkey among nursing and medical students revealed that factors such as age (26 and over), female gender, good economic status, being registered at the faculty of medicine, being in a higher year of study, having sexual experience, and recommending HPV vaccination were associated with higher knowledge scores on HPV, HPV testing, and HPV vaccination (27). Therefore, interventions aimed at improving HPV knowledge and promoting self-sampling may need to consider both general population trends and the unique characteristics of subpopulations.

In this study, higher knowledge scores and higher education levels were associated with a positive attitude towards HPV and self-sampling. On the other hand, the factors influencing attitude towards self-sampling compared to conventional Pap smears among multiethnic Malaysian women were age, ethnicity, and previous Pap smear experience (28). Additionally, in a study focused on a multi-ethnic Asian female population, influencing factors towards the attitude of preference for vaginal self-sampling for HPV testing included higher household income, full-time employment status, not having undergone a Pap test, convenience, and women’s confidence in performing the self-sampling (15). These findings indicate that various factors influence attitudes towards HPV and self-sampling, emphasizing the importance of targeted strategies for promoting cervical screening.

Factors influencing practice varied across different studies and populations. In this study, higher knowledge and attitude scores were associated with good practice. Additionally, being married, engaging in sexual activity, and having a history of HPV infection were also factors associated with favorable practices. However, in a study with female university students in South Africa, knowledge about HPV testing did not positively impact screening practice (19). Factors influencing practice in the United Kingdom included self-efficacy, education level, perceived importance of HPV, knowledge about self-sampling, confidence in self-sampling ability and results, and concerns about sample contamination and identity theft (29). In Gondar, Ethiopia, factors influencing practice encompassed husband disapproval, social influence, lack of knowledge and health education, religious influence, fear of self-sampling, and difficulties with sample collectors and instructions (30). The findings highlight the complex nature of factors influencing HPV self-testing. Understanding these factors is essential for developing targeted interventions and enhancing HPV screening programs.

In this study, higher knowledge and attitude scores were inversely associated with self-sampling for HPV testing. Conversely, belonging to a minority ethnic group and having multiple sexual partners were associated with a higher likelihood of self-sampling. The apparent contradiction between higher knowledge and attitude scores predicting good practice, while lower knowledge and attitude scores are associated with self-sampling, may be due to the fact that individuals with higher scores may perceive traditional methods as more reliable, whereas individuals with lower scores may be more open to considering self-sampling as a convenient and accessible alternative. A study conducted in Geneva adds further relevance by highlighting that women with higher education and professional occupations still prefer clinician sampling, possibly due to their perceived lack of expertise in self-sampling (31). These findings underscore the importance of considering individual preferences and addressing concerns when designing effective HPV screening programs and interventions.

Despite the insightful findings, this study carries some limitations. Firstly, the study’s cross-sectional design inhibits the ability to infer causality between the observed variables and the KAP scores. In addition, the use of self-reported questionnaires may introduce recall bias, social desirability bias, or misunderstandings due to the language or complexity of the questionnaire. The results might also not be generalizable to other populations given the specific demographics of the study participants. Importantly, our research design did not include a sample size calculation nor an explicit strategy to address potential biases, which could affect the robustness and reliability of the findings. Furthermore, the study did not control for some potential confounding factors such as access to healthcare services or the influence of healthcare providers’ advice. The lack of qualitative data may also limit the depth of understanding about the reasons behind certain attitudes and practices. Lastly, the study did not explore the impact of media exposure or health education on the KAP scores, which could play a critical role in shaping knowledge, attitudes, and practices towards HPV and self-sampling.

## Conclusion

In conclusion, our study in Shanghai, China, reveals significant disparities in KAP scores regarding HPV and self-sampling among different demographic groups. While individuals in urban areas and with higher education levels generally exhibited better knowledge, certain misconceptions and concerns regarding self-sampling were prevalent, particularly among married individuals and those with lower knowledge scores. Targeted educational interventions are needed to address these gaps and promote informed decision-making regarding HPV screening. Furthermore, our findings underscore the importance of understanding the complex interplay between knowledge, attitudes, and practices to tailor interventions effectively. By addressing these challenges, we can enhance cervical cancer prevention efforts and reduce the burden of HPV-related diseases in our community.

## Data availability statement

The original contributions presented in the study are included in the article/Supplementary material, further inquiries can be directed to the corresponding authors.

## Ethics statement

The studies involving humans were approved by Shanghai Pudong Hospital Medical Ethics Committee(WZ-019). The studies were conducted in accordance with the local legislation and institutional requirements. Written informed consent for participation was not required from the participants or the participants' legal guardians/next of kin in accordance with the national legislation and institutional requirements.

## Author contributions

JS: Conceptualization, Writing – original draft, Writing review & editing. HK: Writing – original draft, Writing review & editing. CJ: Resources, Formal analysis, Writing original – draft, Writing review & editing. HS: Formal analysis, Writing original – draft, Writing review & editing. HH: Formal analysis, Writing original – draft, Writing review & editing. JZ: Project administration, Writing original – draft, Writing review & editing. LC: Resources, Writing original – draft, Writing review & editing. YW: Resources, Writing original – draft, Writing review & editing. JG: Methodology, Supervision, Writing original – draft, Writing review & editing. YD: Writing original - draft, Writing – review & editing, Funding acquisition.

## References

[1] Okunade KS. Human papillomavirus and cervical cancer. J Obstet Gynaecol. (2020) 40:602–8. doi: 10.1080/01443615.2019.1634030, PMID: 31500479 PMC7062568

[2] Bhat D. The ‘Why and How’of cervical cancers and genital HPV infection. Cytojournal. (2022) 19:22. doi: 10.25259/CMAS_03_03_2021, PMID: 35510113 PMC9063509

[3] de Sanjose S Brotons M Pavon MA. The natural history of human papillomavirus infection. Best Pract Res Clin Obstet Gynaecol. (2018) 47:2–13. doi: 10.1016/j.bpobgyn.2017.08.015 28964706

[4] Ramakrishnan S Partricia S Mathan G. Overview of high-risk HPV's 16 and 18 infected cervical cancer: pathogenesis to prevention. Biomed Pharmacother. (2015) 70:103–10. doi: 10.1016/j.biopha.2014.12.041, PMID: 25776487

[5] Dahlstrom KR Day AT Sturgis EM. Prevention and screening of HPV malignancies. Semin Radiat Oncol. (2021) 31:297–308. doi: 10.1016/j.semradonc.2021.02.011, PMID: 34455985 PMC10124108

[6] Berman TA Schiller JT. Human papillomavirus in cervical cancer and oropharyngeal cancer: one cause, two diseases. Cancer. (2017) 123:2219–29. doi: 10.1002/cncr.30588, PMID: 28346680

[7] Shiraz A Crawford R Egawa N Griffin H Doorbar J. The early detection of cervical cancer. The current and changing landscape of cervical disease detection. Cytopathology. (2020) 31:258–70. doi: 10.1111/cyt.12835, PMID: 32301535

[8] Holcakova J Bartosik M Anton M Minar L Hausnerova J Bednarikova M. New trends in the detection of gynecological precancerous lesions and early-stage cancers. Cancers. (2021) 13:6339. doi: 10.3390/cancers13246339, PMID: 34944963 PMC8699592

[9] Randall TC Salicrup LA Luciani S Trimble EL. HPV testing in resource-limited settings: how can we reach the next level of cervical cancer screening in Latin America and the Caribbean?: Oxford University Press. Oncologist. (2015) 20:1101–4. doi: 10.1634/theoncologist.2015-0201, PMID: 26330459 PMC4591950

[10] Mekuria SF Timmermans S Borgfeldt C Jerkeman M Johansson P Linde DS. HPV self-sampling versus healthcare provider collection on the effect of cervical cancer screening uptake and costs in LMIC: a systematic review and meta-analysis. Syst Rev. (2023) 12:103. doi: 10.1186/s13643-023-02252-y, PMID: 37349822 PMC10286394

[11] Costa S Verberckmoes B Castle PE Arbyn M. Offering HPV self-sampling kits: an updated meta-analysis of the effectiveness of strategies to increase participation in cervical cancer screening. Br J Cancer. (2023) 128:805–13. doi: 10.1038/s41416-022-02094-w, PMID: 36517552 PMC9977737

[12] Di Gennaro G Licata F Trovato A Bianco A. Does self-sampling for human papilloma virus testing have the potential to increase cervical cancer screening? An updated meta-analysis of observational studies and randomized clinical trials. Front Public Health. (2022) 10:1003461. doi: 10.3389/fpubh.2022.1003461, PMID: 36568753 PMC9773849

[13] Gerend MA Shepherd MA Kaltz EA Davis WJ Shepherd JE. Understanding women's hesitancy to undergo less frequent cervical cancer screening. Prev Med. (2017) 95:96–102. doi: 10.1016/j.ypmed.2016.11.028, PMID: 27932055

[14] Saville M Hawkes D McLachlan E Anderson S Arabena K. Self-collection for under-screened women in a National Cervical Screening Program: pilot study. Curr Oncol. (2018) 25:27–32. doi: 10.3747/co.25.3915, PMID: 29507492 PMC5832287

[15] Khoo SP Lim WT Rajasuriar R Nasir NH Gravitt P Woo YL. The acceptability and preference of vaginal self-sampling for human papillomavirus (HPV) testing among a multi-ethnic Asian female PopulationAcceptability and preference of self-sampling HPV testing. Cancer Prev Res. (2021) 14:105–12. doi: 10.1158/1940-6207.CAPR-20-0280, PMID: 32917643

[16] Zhao Y Liao Q Mi X Li M Zhao C Cui S. Survey of the acceptance status of HPV self-sampling screening in female population for cervical cancer. Zhonghua Fu Chan Ke Za Zhi. (2019) 54:312–7. doi: 10.3760/cma.j.issn.0529-567x.2019.05.005, PMID: 31154712

[17] Qu S-X Ni Y-H Qin J-F Chen X-Y Wu W-L Zhang W-C. Experience and acceptability for HPV self-sampling among women in Jiangsu province, China: a cross-sectional survey. J Obstet Gynaecol. (2023) 43:2204942. doi: 10.1080/01443615.2023.2204942, PMID: 37129887

[18] Lee M Kang BA You M. Knowledge, attitudes, and practices (KAP) toward COVID-19: a cross-sectional study in South Korea. BMC Public Health. (2021) 21:295. doi: 10.1186/s12889-021-10285-y, PMID: 33546644 PMC7863060

[19] Eche MT Vermaak K. Knowledge, attitude and practice of female university students regarding human papillomavirus and self-sampling in KwaZulu-Natal, South Africa: a cross-sectional survey. BMC Womens Health. (2022) 22:1–58. doi: 10.1186/s12905-022-01634-z 35246111 PMC8895517

[20] He L He J. Attitudes towards HPV self-sampling among women in Chengdu, China: a cross-sectional survey. J Med Screen. (2020) 27:201–6. doi: 10.1177/0969141319895543, PMID: 31896295

[21] Wang J Jing R Lai X Zhang H Lyu Y Knoll MD. Acceptance of COVID-19 vaccination during the COVID-19 pandemic in China. Vaccine. (2020) 8:482. doi: 10.3390/vaccines8030482, PMID: 32867224 PMC7565574

[22] Kops NL Hohenberger GF Bessel M Horvath JDC Domingues C Maranhão AGK. Knowledge about HPV and vaccination among young adult men and women: results of a national survey. Papillomavirus Res. (2019) 7:123–8. doi: 10.1016/j.pvr.2019.03.003, PMID: 30885798 PMC6426699

[23] Qayum MO Billah MM Akhter R Flora MS. Women’s knowledge, attitude and practice on cervical Cancer and its screening in Dhaka, Bangladesh. Asian Pac J Cancer Prevent. (2021) 22:3327–35. doi: 10.31557/APJCP.2021.22.10.3327 PMC8858246 34711010

[24] Grigore M Teleman SI Pristavu A Matei M. Awareness and knowledge about HPV and HPV vaccine among Romanian women. J Cancer Educ. (2018) 33:154–9. doi: 10.1007/s13187-016-1130-2, PMID: 27830570

[25] Murchland AR Gottschlich A Bevilacqua K Pineda A Sandoval-Ramírez BA Alvarez CS. HPV self-sampling acceptability in rural and indigenous communities in Guatemala: a cross-sectional study. BMJ Open. (2019) 9:e029158. doi: 10.1136/bmjopen-2019-029158, PMID: 31662358 PMC6830827

[26] Zibako P Tsikai N Manyame S Ginindza TG. Knowledge, attitude and practice towards cervical cancer prevention among mothers of girls aged between 9 and 14 years: a cross sectional survey in Zimbabwe. BMC Womens Health. (2021) 21:1–13. doi: 10.1186/s12905-021-01575-z 34930221 PMC8691087

[27] Akalin A. Knowledge and attitude towards human papillomavirus and its vaccination and affecting factors among nursing and medical students: a questionnaire study. J Obstet Gynaecol. (2022) 42:3315–21. doi: 10.1080/01443615.2022.2124851 36129449

[28] Ma'som M Bhoo-Pathy N Nasir NH Bellinson J Subramaniam S Ma Y. Attitudes and factors affecting acceptability of self-administered cervicovaginal sampling for human papillomavirus (HPV) genotyping as an alternative to pap testing among multiethnic Malaysian women. BMJ Open. (2016) 6:e011022. doi: 10.1136/bmjopen-2015-011022, PMID: 27491667 PMC4985871

[29] Williams D Davies M Fiander A Farewell D Hillier S Brain K. Women's perspectives on human papillomavirus self-sampling in the context of the UK cervical screening programme. Health Expect. (2017) 20:1031–40. doi: 10.1111/hex.12544, PMID: 28186384 PMC5600225

[30] Megersa BS Bussmann H Bärnighausen T Muche AA Alemu K Deckert A. Community cervical cancer screening: barriers to successful home-based HPV self-sampling in Dabat district, North Gondar, Ethiopia. A qualitative study. PLoS One. (2020) 15:e0243036. doi: 10.1371/journal.pone.0243036, PMID: 33306681 PMC7732077

[31] Sormani J Kenfack B Wisniak A Moukam Datchoua A Lemoupa Makajio S Schmidt NC. Exploring factors associated with patients who prefer clinician-sampling to HPV self-sampling: a study conducted in a low-resource setting. Int J Environ Res Public Health. (2021) 19:54. doi: 10.3390/ijerph19010054, PMID: 35010314 PMC8744711

