# Supplementary material for: Knowledge, attitudes, and practices of human papillomavirus and self-sampling among adult women: a cross-sectional study
Source: Front Public Health. 2024 Jun 4;12:1377343. doi: 10.3389/fpubh.2024.1377343 (PMC11184059; doi:10.3389/fpubh.2024.1377343)
Supplement: Supplementary file 1 [file Table_1.pdf]

Supplementary Table 1 Baseline characteristics and KAP scores

Variables	N (%)	Knowledge score		Attitude score		Practice score	
		Mean ± SD	P	Mean ± SD	P	Mean ± SD	P
**Total**	1843	10.09±5.60		26.76±3.80		6.24±2.20	
**Age (years old)**			0.736		0.004		<0.001
18-30	403(21.87)	11(5, 14)		27(24, 30)		6(4, 8)	
30-35	452(24.53)	11(6, 15)		27(24, 29)		6(6, 8)	
35-40	375(20.35)	11(6, 15)		26(24, 29)		6(6, 8)	
40-45	311(16.87)	11(6, 15)		27(24, 29)		6(6, 8)	
45 and above	302(16.39)	12(6, 15)		26(24, 29)		6(6, 8)	
**Residence**			<0.001		0.026		<0.001
Rural	475(25.77)	7(2, 12)		26(24, 28)		6(4, 8)	
Urban	741(40.21)	13(8, 16)		27(24, 30)		6(6, 8)	
Suburban	627(34.02)	12(7, 15)		27(24, 29)		6(6, 8)	
**Ethnicity**			0.021		0.912		0.019
Han	1789(97.07)	11(6, 15)		26.76±3.82		6(6, 8)	

Minority	54(2.93)	6(4, 14)		26.81±3.30		6(4, 8)	
**Education**			<0.001		<0.001		<0.001
Primary and below	32(1.74)	2(0, 6)		25.38±3.18		4.88±2.78	
Middle School	305(16.55)	6(2, 10)		25.88±3.28		5.70±2.61	
High school / Technical secondary school	244(13.24)	8(3, 12)		26.29±3.56		5.88±2.54	
Junior college / Undergraduate	1105(59.96)	12(8, 15)		26.96±3.93		6.45±1.98	
Postgraduate and above	157(8.52)	16(12, 17)		28.08±3.78		6.69±1.79	
**Employment Status**			<0.001		<0.001		<0.001
Employed	1375(74.61)	10.86±5.36		26.85±3.83		6(6, 8)	
Unemployed	44(2.39)	7.43±5.45		25.82±3.22		6(6, 8)	

Retired	47(2.55)	9.26±5.68		25.60±3.27		6(4, 8)	
Freelance	140(7.60)	7.54±5.72		26.95±4.07		6(4, 8)	
Housewife	133(7.22)	6.33±5.25		25.82±3.69		6(4, 8)	
Student	45(2.44)	13.27±3.46		28.69±3.06		6(6, 8)	
Other	59(3.20)	6.93±5.54		26.41±3.17		6(4, 8)	
**Household income per capita (Yuan)**			<0.001		<0.001		<0.001
<2000	90(4.88)	3(0, 8)		25.61±3.36		6(4, 8)	
2000-5000	411(22.30)	9(4, 13)		26.22±3.66		6(4, 8)	
5000-10000	721(39.12)	11(6, 15)		26.87±3.68		6(6, 8)	
10000-20000	431(23.39)	13(8, 16)		27.06±4.13		6(6, 8)	
>20000	190(10.31)	13(8, 16)		27.34±3.77		6(6, 8)	
**Marital status**			<0.001		0.058		<0.001
Unmarried	171(9.28)	13(7, 16)		27.41±3.74		6(6, 8)	

Married	1596(86.60)	11(6, 15)		26.70±.3.81		6(6, 8)	
Divorced / Widowed	76(4.12)	11(6, 15)		26.53±3.60		6(4, 8)	
**Sexual life**			0.963		0.862		<0.001
Yes	1709(92.73)	14(12, 16)		26.75±3.82		8(6, 8)	
No	134(7.27)	10(5, 14)		26.81±3.50		6(6, 8)	
**The number of sexual partners**			0.863		0.502		0.685
1	1527(82.85)	10.10±5.57		26.72±3.81		6(6, 8)	
2	118(6.40)	9.88±5.75		26.82±4.16		6(4, 8)	
≥3	64(3.47)	10.58±5.21		27.45±3.49		7(4.5, 8)	
**Family history of cervical cancer**			<0.001		0.178		0.008
Yes	31(1.68)	12.19±4.83		27.71±4.43		8(6, 8)	
No	1709(92.73)	10.26±5.56		26.77±3.80		6(6, 8)	

Unclear	103(5.59)	6.78±5.42		26.30±3.61		6(4, 8)	
**History of HPV infection**			<0.001		0.021		<0.001
Yes	195(10.58)	14(12, 16)		27.5(24.75, 29)		8(6, 8)	
No	1648(89.42)	10(5, 14)		26(24, 29)		6(6, 8)	
**Smoking status**			0.013		0.347		0.873
Never smoked	1737(94.25)	11(6, 15)		26.79±3.77		6(6, 8)	
Former smoker	75(4.07)	8(4, 13.5)		26.40±4.33		6(5, 8)	
Current smoker	31(1.68)	6(4, 14)		25.97±4.06		6(6, 8)	
